# The Bedding and Linen Department—VI

**Published:** 1908-06-06

**Authors:** 


### Nursing Administration.

### THE BEDDING AND LINEN DEPARTMENT.

### VI.—THE COLLECTION OF SOILED LINEN.

When the enormous number of articles sent to the wash every week in a large institution is considered, it becomes evident that unless a good system is in force for collecting and returning the linen serious confusion, leading to perpetual losses, must result. To rid the ward expeditiously of soiled linen the " shoot " system was devised some years ago, and for a time this was considered " le dernier cri " in hospital architecture. Every ward was fitted with a shoot which led down to a room in the basement, where the linen rested till sorted and despatched to the wash. The shoot undoubtedly had its conveniences, but it has lost favour on more than one account. Its chief defect—a tendency to harbour germs—suffices, however, in the present day to put it out of court altogether, and in no new hospital would it be inserted. Each ward is now commonly provided with an outside soiled-linen closet, and here the linen is deposited as removed from the ward until it is despatched in bags, baskets, or other containers. In very large institutions the linen is often despatched to the laundry every day; in others two or three times a week, and in small hospitals once a week may suffice. Whatever the rule may be, it is the duty of the ward sister to prepare a list with counterfoil of all she sends down to be washed, and it greatly facilitates her labours if these lists are differently tinted according to the day. Monday's list can be made out on blue paper, Wednesday's on red, Friday's on yellow. And thus she knows at a glance which sending is being returned to her. Septic articles are, of course, not retained with the rest of the soiled linen, but are despatched in a proper receptacle to be disinfected before they reach the laundry. These articles are, however, to be added to her list, under a separate heading, as they will be returned with the rest of the clean linen. The sister's duty towards the soiled linen ceases when she has checked over the wash for the day or week, has made out the list to go with it, and filed away the counterfoil. Down below it falls under the sway of the housekeeper or linen sister. In hospitals where there is no private laundry there must be a room set apart for the reception of the soiled linen, when sent down from the wards, where the lists can be checked and the totals entered in the register for the laundry. Here also the soiled linen collected by the head housemaid from various departments in the institution must be checked, and care taken that all towels, for instance, given out for lavatories, kitchen cloths, etc., have been given in, according to lists supplied. It is the duty of the linen sister also to check the disinfected linen, taking steps to see that the process has been properly carried out before it is sent away to the laundry.

Where there is a private laundry, however, all this can be more properly carried out in the laundry itself. In that case the linen is received by the laundry superintendent, or head laundry maid, just as it comes from the wards and other departments of the hospital, and is entered into a register under the various headings with totals, so that it is possible to tell on any given day exactly how much linen was sent from each ward in the house. The following is a specimen of the form in which this register is kept:—

Laundry Register.

*Date*..................

| — | Ward A | Ward B &c. | Out-patients | Theatre | House | Total |
|---|---|---|---|---|---|---|
| Counterpanes ... | | | | | | |
| Blankets ... | | | | | | |
| Sheets ... ... | | | | | | |
| Draw-sheets ... | | | | | | |
| Tablecloths ... | | | | | | |
| &c., &c. ... | | | | | | |

On the precision with which this register is maintained the successful organisation of the home laundry may be said to depend.

The process has now to be reversed and the clean linen got back safely to the wards. The public laundry will return the linen in bulk; hence the necessity for a sorting-room in which it can be checked through by the linen sister and sorted for the different wards and departments from whence it was issued. In such a sorting-room should be facilities for airing the sheets and pillow cases before they are returned to the wards if not sent back perfectly dry. Where the private laundry exists all this may, as in the case of the soiled linen, be done on the laundry premises. The linen is aired and sorted before it is sent over to the hospital, and the laundry maid who takes it round may be charged with the duty of checking through the amounts returned, by aid of the counterfoil, with the sister or someone appointed by her for this duty. An exact memorandum on a form with counterfoil, supplied for the purpose, must now be made of any missing articles, and the counterfoil retained. There remains the duty of arranging the linen on the shelves and setting all in exact order.

One point deserves more attention than it often receives. The atmospheric conditions in this country constitute a serious danger in establishments where small wards often remain untenanted and without fires for several weeks at a time. A damp fog following a frost, or a sea fret such as is common on the South and East Coasts, will leave a trail of moisture on every coverlet, blanket, and sheet exposed to its influence, and will even soak through the head of the mattress near an open window. In London we have seen beds, left for only a week or two unoccupied, where every article turned wet and slimy when spread out before the fire, and the mattress steamed at every exposed point of the surface. Surprising results may sometimes be obtained by placing the coverlets from occupied beds in ordinary wards round the fire after such weather as we have described.

